# Supplementary material for: The Kinetic and Analytical Aspects of Enzyme Competitive Inhibition: Sensing of Tyrosinase Inhibitors
Source: Biosensors (Basel). 2021 Sep 8;11(9):322. doi: 10.3390/bios11090322 (PMC8471001; doi:10.3390/bios11090322)
Supplement: Supplementary file 1 [file biosensors-11-00322-s001.zip › biosensors-1336695-supplementary.pdf]

**Supplementary materials**

# The Kinetic and Analytical Aspects of Enzyme Competitive Inhibition: Sensing of Tyrosinase Inhibitors

Raouia Attaallah, Aziz Amine*

*Faculty of Sciences and Techniques, Hassan II University of Casablanca, PA 146 Mohammedia, 20800, Morocco*

* Corresponding author: Tel.: +212 661 455198, Fax +212 523 315353. a.amine@univh2m.ac.ma

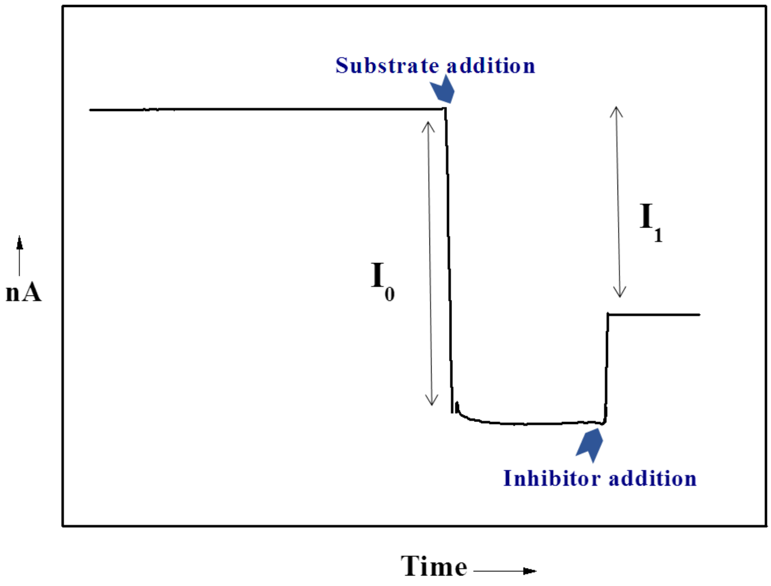


**Figure S1.** Typical inhibition biosensor response in the absence ($I_0$), and in the presence of inhibitor ($I_1$).

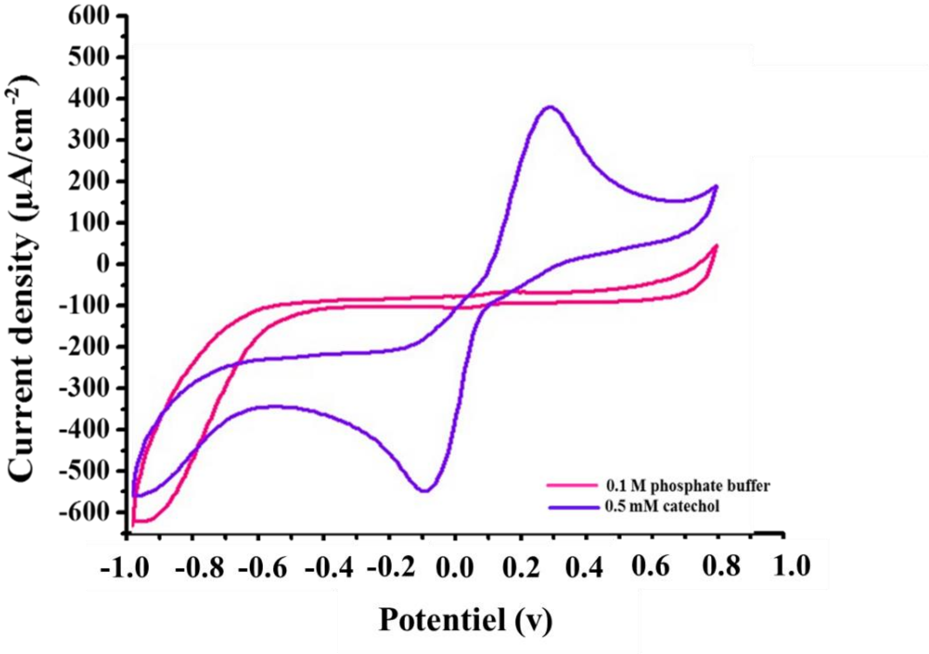


**Figure S2**. Cyclic voltammetry of 0.5 mM catechol with Tyr-CBPE in 0.1 M phosphate buffer pH 6.8, scan rate of 50 mV·s$^{-1}$.

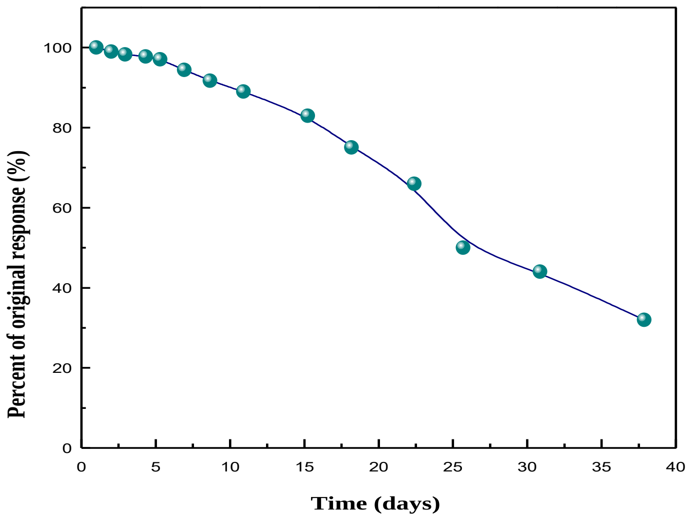


**Figure S3.** Relative response of tyrosinase as a function of the storage time. In the presence of 20 µM catechol in 0.1 M phosphate buffer, pH 6.8. Applied potential -0.15 V vs Ag/AgCl.
